# CDK12 Activity-Dependent Phosphorylation Events in Human Cells

**DOI:** 10.3390/biom9100634

**Published:** 2019-10-22

**Authors:** Bartlomiej Bartkowiak, Christopher M. Yan, Erik J. Soderblom, Arno L. Greenleaf

**Affiliations:** 1 Department of Biochemistry, Duke Med. Ctr., Durham, NC 27710, USA; bartjbartkowiak@gmail.com (B.B.); cmartinyan@gmail.com (C.M.Y.); 2 Proteomics Core Facility, Duke Med. Ctr., Durham, NC 27710, USA; erik.soderblom@duke.edu

**Keywords:** analog-sensitive CTD kinase, transcription, CTD, RNA polymerase II, RNA processing, mRNA nuclear export, nuclear pore, mESC undifferentiated state, XPC, TPR, 1-NM-PP1

## Abstract

We asked whether the C-terminal repeat domain (CTD) kinase, CDK12/CyclinK, phosphorylates substrates in addition to the CTD of RPB1, using our CDK12^analog-sensitive^ HeLa cell line to investigate CDK12 activity-dependent phosphorylation events in human cells. Characterizing the phospho-proteome before and after selective inhibition of CDK12 activity by the analog 1-NM-PP1, we identified 5,644 distinct phospho-peptides, among which were 50 whose average relative amount decreased more than 2-fold after 30 min of inhibition (none of these derived from RPB1). Half of the phospho-peptides actually showed >3-fold decreases, and a dozen showed decreases of 5-fold or more. As might be expected, the 40 proteins that gave rise to the 50 affected phospho-peptides mostly function in processes that have been linked to CDK12, such as transcription and RNA processing. However, the results also suggest roles for CDK12 in other events, notably mRNA nuclear export, cell differentiation and mitosis. While a number of the more-affected sites resemble the CTD in amino acid sequence and are likely direct CDK12 substrates, other highly-affected sites are not CTD-like, and their decreased phosphorylation may be a secondary (downstream) effect of CDK12 inhibition.

## 1. Introduction

Human CDK12, a tumor suppressor for ovarian and other cancers [1,2], is the catalytic subunit of the protein kinase complex CDK12/CyclinK [3,4]. CDK12/CyclinK has been shown to phosphorylate the C-terminal repeat domain (CTD) of RPB1, the largest subunit of RNA polymerase II [3,5,6]. CDK12 is the ortholog of the yeast protein Ctk1, the catalytic subunit of the major yeast transcription elongation-phase CTD kinase, CTDK-I [7,8]. Yeast CTDK-I has been shown to play roles in pre-mRNA processing [9,10,11], DNA damage-induced transcription [12], chromatin modification [13,14], mRNP formation & nuclear export [15,16], responses to DNA damage [17], mitotic homologous recombination [17], and even translation [18]. In vertebrates, CDK12 has a less-studied paralog, CDK13.

By RNA-mediated knock-down or by chemical inhibition of catalytic activity, metazoan CDK12 has been implicated in several processes in vivo, and as anticipated these overlap with Ctk1-influenced processes in yeast. For example, mammalian CDK12 has been connected with expression of genes involved in DNA damage responses [4,19], it has been implicated in mRNA 3′ end formation [20,21], and CDK12 and its paralog CDK13 have been implicated in RNA processing and in expression of RNA processing factors [19]. Related to these latter observations, CDK12 and CDK13 have been shown biochemically to bind a number of RNA processing factors [3,19]. In addition, CDK12 and CDK13 have been shown to be important for mouse Embryonic Stem Cells (ESC) self-renewal [22] and for early steps in development [23]. In the fruit fly, CDK12 has been shown to be needed for glial-specific splicing of Neurexin IV [24].

Overall, a picture is emerging in which CDK12 plays major roles in transcription, RNA processing and genome stability. Consistent with a role in genome stability, CDK12 was identified as a likely tumor suppressor for ovarian cancer several years ago [1,2], and evidence continues to accumulate linking CDK12 alterations to ovarian and other types of cancer [25,26]. Of major importance are two recent studies that demonstrate/confirm CDK12 is a tumor suppressor for ovarian and prostate cancers and go on to show that bi-allelic loss of CDK12 in ovarian or prostate tumor cells leads to large numbers of tandem duplications spread throughout the genome [27,28]. This striking DNA phenotype may help explain why loss of CDK12 is tumorigenic; however, the underlying molecular events connecting CDK12 loss with the DNA duplications have yet to be worked out. 

Phenotypes caused by knocking-down or inhibiting the activity of CDK12 are usually attributed to changes in phosphorylation of the CTD, but it is possible that this enzyme also phosphorylates other protein substrates and that altered phosphorylation of these non-CTD substrates leads to some of the observed phenotypes. Along these lines, other CTD kinases have been shown to perform functionally meaningful phosphorylation of non-CTD substrates. Thus, CDK9 phosphorylates SPT5, facilitating transformation of a paused pol II complex into an actively elongating one [29]. In fact, CDK12 itself has recently been implicated in phosphorylation of several non-CTD substrates. For example, CDK12/CyclinK appears necessary for phosphorylation of Cyclin E1 that helps to regulate assembly of pre-replicative complexes during G1 [30]. Also, CDK12 was shown to phosphorylate translation factor 4E-BP1, thereby regulating translation of a subset of mRNAs, notably encoding proteins involved in cell division [31], and CDK12 has been implicated in phosphorylating certain RNA processing factors [32]. 

We hypothesized that additional non-CTD substrates of CDK12 exist, and we felt that our CDK12^analog-sensitive^ HeLa cell line represented a good system for finding such substrates. This cell line (HeLa “CDK12^as^”) expresses only an analog-sensitive, selectively-inhibitable version of CDK12 [5]. We initially used the CDK12^as^ cell line to test gross effects of CDK12 inhibition, demonstrating that selective inhibition of CDK12 catalytic activity leads to altered CTD phosphorylation, as expected. Interestingly, inhibiting CDK12 also leads to relatively quick growth arrest, not necessarily an expected result [5]. 

To discover potential non-CTD substrates of CDK12, we analyzed changes in the phospho-proteome of HeLa CDK12^as^ cells following selective inhibition of CDK12. When the adenosine analog 1-NM-PP1 is added to growing CDK12^as^ cells, CDK12 activity is rapidly inhibited, as reflected in changes of CTD phosphorylation patterns observable in 15 min or less [5]. If CDK12 also phosphorylates non-CTD proteins, the level of their phosphorylation at CDK12 phosphorylation sites is likewise expected to decrease after CDK12 inhibition. To check this expectation, we used mass spectrometry (MS) approaches to analyze the phospho-proteome of CDK12^as^ cells grown without or with addition of the inhibitory analog, 1-NM-PP1. To maximize the chances of identifying direct substrates of CDK12, rather than downstream, indirect, substrates, we carried out the inhibition for only a short time (30 min). As a type of control experiment, to uncover off-target effects of 1-NM-PP1, we also assessed changes in the phospho-proteome of CDK12^wild-type^ cells after analog addition.

## 2. Materials and Methods

### 2.1. Cell Growth

The HeLa CDK12^as^ cell line [5] was grown in DMEM with 10% FBS at 37 °C and 5% CO_2_. Six 10 cm plates were cultured to a cell density of ~80%. At time 0, 1-NM-PP1 or DMSO was added to each of 3 plates to a final concentration of 10 µM 1-NM-PP1 (20 µL of 20 mM 1-NM-PP1 in DMSO stock solution was added to 40 mL of fresh, prewarmed, media and mixed; the growth media on each plate was removed and replaced with 10 mL of this solution). Control plates were treated with DMSO-containing media. After 30 min, cells were harvested by trypsinization (all buffers used subsequent to treatment, including PBS, Trypsin, and media used for neutralization, contained 10 uM 1-NM-PP1 or the equivalent volume of DMSO). The cells were concentrated by centrifugation (500× *g*, 3 min, at 4 °C), supernatant was removed, and the pellets snap frozen in liquid nitrogen; cell pellets were stored at −80 °C.

### 2.2. Proteomic Analyses

#### 2.2.1. Sample Preparation

To each pellet, 200 µL of 50 mM ammonium bicarbonate was added, pH 8.0 with 8M urea. Samples were subjected to three rounds of probe sonication for 5s each with an energy setting of 30%. Samples were then centrifuged at 12,000× *g* at 4 °C for 5 min. Protein concentrations were determined by Bradford assay on the supernatant in duplicate (2 µL each assay). Total protein concentrations ranged from 3.9 mg/mL to 4.3 mg/mL with total protein quantities ranging from 786 µg to 862 µg. 

Subsequently, 300 ug of each sample was removed and normalized to 3.93 mg/mL with 50 mM ammonium bicarbonate containing 8M urea. To each sample, 45 pmol of Casein_Bovine was added (30 fmol/ug lysate) as an intact protein and as an internal standard for phosphopeptide enrichment. All samples were then reduced for 20 min at 80 °C with 10 mM dithiothreitol and alkylated for 40 min at room temperature with 22 mM iodoacetamide. Samples were then diluted to 1.6M urea with 50 mM ammonium bicarbonate. Trypsin was added to a 1:50 ratio (enzyme to total protein) and allowed to proceed for 18 h at 37 °C. Samples were then acidified with 0.2% TFA (pH 2.5) and subjected to C18 SPE cleanup (Sep-Pak, 50 mg bed, Waters Corp., Taunton, MA, USA). Following elution, all samples were frozen and then lyophilized to dryness. Details regarding sample preparation protocols are available in Supplementary File I Table S1.

#### 2.2.2. Phospho-Peptide Enrichment

Lyophilized peptides were resuspended in 150 µL of 1M glycolic acid in 80% acetonitrile/1% TFA. Phosphopeptide enrichments were done using 200 µL GL Bioscience TiO2 spin tips as per a standard protocol established in the DPCF: http://www.genome.duke.edu/cores/proteomics/sample-preparation/documents/GL_SpinColumnProtocol_bmr_ejs_mt_061713.pdf Eluted phosphopeptides were then subjected to stage tip C18 cleanups and then brought to dryness with lyophilization. Samples were then resuspended in 20 uL of 10 mM citric acid in 0.1% TFA/2% acetonitrile containing 10 fmol/uL yeast_ADH. To create a “QC pool” sample to assess analytical reproducibility, 5 uL of each sample was removed and pooled.

#### 2.2.3. Quantitative Analysis of HeLa Phosphoproteome

Quantitative LC/MS/MS was performed on 4 uL of each phosphopeptide enriched sample, using a nanoAcquity UPLC system (Waters Corp., Taunton, MA, USA) coupled to a Thermo QExactive Plus high resolution accurate mass tandem mass spectrometer (ThermoFisher, Waltham, Massachusetts, MA, USA) via a nanoelectrospray ionization source. Briefly, the sample was first trapped on a Symmetry C18 300 mm × 180 mm trapping column (5 μL/min at 99.9/0.1 *v/v* water/acetonitrile) (Waters Corp., Taunton, MA, USA), after which the analytical separation was performed using a 1.7 um Acquity BEH130 C18 75 mm × 250 mm column (Waters Corp, Taunton, MA, USA) using a 5-min hold at 3% acetonitrile with 0.1% formic acid and then a 90- min gradient of 3 to 30% acetonitrile with 0.1% formic acid at a flow rate of 400 nanoliters/minute (nL/min) with a column temperature of 55C. Data collection on the QExactive Plus mass spectrometer was performed in a data-dependent acquisition (DDA) mode of acquisition with a *r* = 70,000 (@ *m/z* 200) full MS scan from *m/z* 375–1600 with a target AGC value of 1e6 ions followed by 10 MS/MS scans at *r*-17,500 (@ *m/z* 200) at a target AGC value of 5e4 ions. A 20s dynamic exclusion was employed to increase depth of coverage. The total analysis cycle time for each sample injection was approximately 2-h.

Sample order of data collection was interwoven between conditions in order to minimize temporal bias, and run order is shown in Supplementary File I Table S2. Following the 9 LC-MS/MS analyses, data were imported into Rosetta Elucidator v3.3 (Rosetta Biosoftware, Inc, Seattle, WA, USA), and all LC-MS/MS runs were aligned based on the accurate mass and retention time of detected ions (“features”) which contained MS/MS spectra using PeakTeller algorithm (Elucidator, Rosetta Biosoftware) and intensity-scaled based on a robust mean (10%) normalization of the identified phosphopeptide features. The relative peptide abundance was calculated based on area-under-the-curve (AUC) of aligned features across all runs. The overall dataset had 71,814 quantified isotope (peptide) groups. Additionally, 395,960 MS/MS spectra were acquired for peptide sequencing by database searching. This MS/MS data were searched against a SwissProt_Human database (https://web.expasy.org/docs/swiss-prot_guideline.html) which also contained a reversed-sequence “decoy” database for false positive rate determination as well as Casein_Bovine as a surrogate internal standard. Database searching was performed within Mascot Server (Matrix Science, Boston, MA, USA) and annotated at a Mascot ion tolerance of 20.0 which resulted in a peptide false discovery rate of 0.47%. Searching allowed variable M (oxidation, +16 Da), and STY (phosphorylation, +80 Da). Searching allowed variable M (oxidation, +16 Da), and STY (phosphorylation, +80 Da). For MS/MS spectra containing multiple matches to different phosphopeptide localizations, the assignment of the highest mascot ion score was used to assign the identification to the corresponding MS1 peak. 

This analysis yielded identifications for 7892 unique non-decoy peptides (The Supplementary File I Table S3), of which 5644 were phosphorylated (Supplementary File I Table S4). These 5644 phosphopeptides corresponded to 2038 phosphoproteins. The phospho-peptides that changed 2-fold or more after CDK12 inhibition are listed in Supplementary File I Table S5.

#### 2.2.4. Assessment of Variability

To assess the technical variability in the phospho-proteomics LC-MS/MS assay, a Sample Pool Quality Control (SPQC) sample was created by pooling a small portion of each individual sample following TiO2 phosphopeptide enrichment. This SPQC sample was acquired as the first, the middle and the last injection in the run order queue with individual biological replicates randomly run between. The SPQC injections were informatically processed identically to the individual biological replicates allowing for analytical variability to be measured for each of the phosphopeptides identified across the entire study. Triplicate measurements of this SPQC sample resulted in an average %CV (relative standard deviation) of 13.6% and a median %CV of 8.5% across all n = 5,644 phosphopeptides. To assess TiO2 enrichment variation, trypsin digested Bovine Casein was spiked into each individual sample prior to TiO2 phosphopeptide enrichment. The %CV of each of 15 unique phosphopeptides from Bovine Casein was 14.7%. To measure analytical variation independent of TiO2 enrichment variation, pre-digested yeast ADH was also spiked post TiO2 enrichment. The average %CV of the 12 measured ADH peptides was 23.0%. To assess biological variation across the same *n* = 5644 phosphopeptides, the intensity-scaled phosphopeptide expression data from all identified phosphopeptides within DMSO control and 1-NM-PP1 inhibitor were calculated to be 18.0% and 18.9%, respectively. All of the processed data for which these metrics were calculated are available in The Supplementary File I Table S4.

## 3. Results

### 3.1. Phosphorylation Events Altered by Inhibitory Analog in CDK12^as^

**Figure 1:**
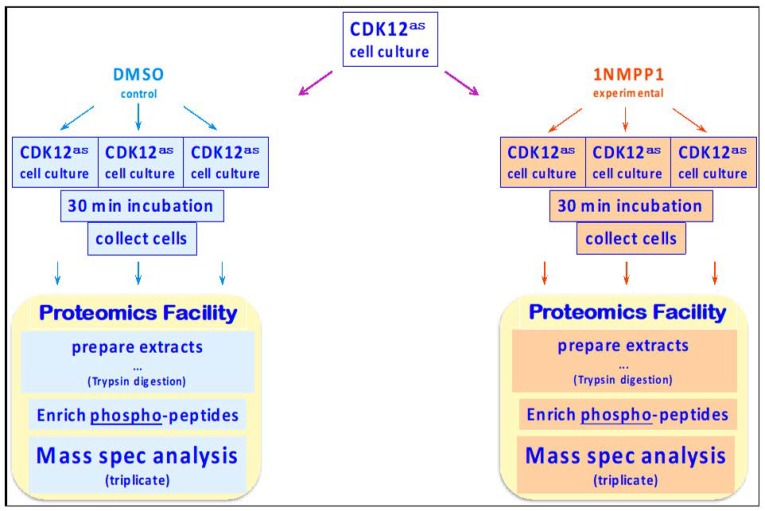
Diagrammatic overview of experimental approach.

Exponentially-growing CDK12^as^ cells were incubated for 30 min, in triplicate, in the absence or presence of the inhibitory analog 1-NM-PP1; then cells were rapidly collected (pelleted by centrifugation) and frozen (Figure 1). The cell pellets were processed to optimally solubilize proteins/peptides for mass spectrometry, the proteins were subjected to Trypsin cleavage, and phospho-peptides (P-peptides) present in the solutions were affinity-enriched using TiO_2_ columns. The P-peptide–enriched samples were analyzed by mass spectrometry techniques to identify, sequence, and obtain relative abundance data on the P-peptides (for details, see Materials & Methods). 

The analysis identified 5,644 total unique P-peptides representing 2,038 proteins (Supplementary File I Table S4). The average relative abundances of each P-peptide in the DMSO- (control) and 1-NM-PP1–treated samples were compared, and P-peptides that changed in amount more than 2-fold after the 30 min exposure to inhibitor were identified. All together, fifty P-peptides (representing 40 proteins) decreased >2-fold in abundance after inhibitor treatment; in contrast, only 3 P-peptides increased >2-fold (Supplementary File I Table S5—note, “WT control experiment” is presented in a later section). Among the group of fifty P-peptides, half actually showed a decrease of 3-fold or more, with a dozen of those decreasing more than 5-fold. Variability in the results was assessed as described in Materials and Methods.

To focus on the most CDK12-dependent P-peptides, we have listed in Table 1 the proteins that yielded P-peptides showing a decrease in amount of >3-fold. The proteins are grouped into five broad categories based on their known or suspected functions (Functional “Keywords”); a protein is listed one time for each peptide derived from it. We see that although these proteins are fairly diverse, a majority of the P-proteins fall into functional categories with known or likely connections to either CDK12 or the phosphoCTD. For example, the largest category contains proteins with functions that center in some way around transcription regulation and “signaling”. Of course, CDK12 is already linked to transcription by virtue of CTD phosphorylation, but the phospho-proteomics results reported here indicate that the phosphorylation status of these non-polymerase proteins also depends on CDK12 activity. The next largest group of proteins function in various phases of pre-mRNA processing. Again, CDK12 has been connected to pre-mRNA processing events either through RNAi-mediated depletion experiments or by co-IP studies (see Introduction); the current results indicate that CDK12 may also affect RNA processing events by mediating, directly or indirectly, the phosphorylation state of certain processing components (see also [32]). Two smaller categories in Table 1 contain proteins that function in aspects of mitosis and “DNA synthesis/repair,” both of which have also been associated with CDK12 and. Finally, two proteins fall into the category “other” and have no previous connection to CDK12. 

Before continuing the discussion of the affected proteins, we point out a noticeable feature of the results: no RPB1 P-peptides (i.e., from the CTD) were identified as changing 2-fold or more (Supplementary File I, Tables S4 and S5). This is mainly due to the distribution of Trypsin cleavage sites in the CTD [33,34]. The first Trypsin cleavage site in the human CTD is in repeat 2, but the next one is not until after repeat 31, and the CTD fragment released by cleavage at these two sites will be 197 residues long, which is too large for the MS analysis. In contrast, the remainder of the CTD contains 8 Lys residues and will be cleaved by Trypsin to produce 9 short peptides, several of which are too small for the analysis [33]. A search through the unfiltered P-peptides reported in our analysis (Supplementary File I, Table S4) did reveal 5 CTD-derived P-peptides from this region, but none displayed a 2-fold difference in abundance between control and inhibitor-treated cells. Thus, our experiment does not provide new information on where CDK12 adds phosphates to the CTD in vivo.

**Figure 2:**
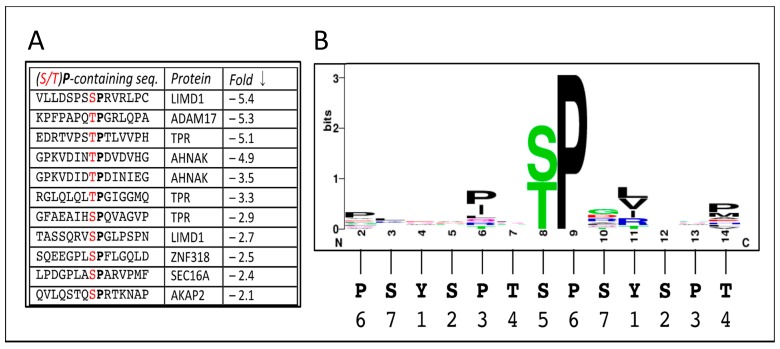
Potential consensus phosphorylation site for CDK12. The phospho-peptides that contain an (S/T)P dipeptide that decreased > 2-fold after CDK12 inhibition (from Supplementary File I, Table S5) are listed in (**A**) (first column), aligned on the phosphorylated S/T residue. The parent protein and the fold decrease are in the second and third columns, respectively. This sequence set was used to find a sequence logo using <weblogo.berkeley.edu>, with the logo range set to 3-14; results in (**B**).

Returning to the proteins whose phosphorylation does change significantly after inhibiting CDK12 activity, we expect several of these to be directly phosphorylated by CDK12/CyclinK. The most likely direct substrates are those with amino acid sequences resembling the CTD consensus sequence (see Figure 2). On the other hand, the majority of the MS-identified phosphorylation sites (P-sites) do not obviously resemble CTD sequences (Supplementary File I, Table S5); nevertheless, some of them may prove to be phosphorylated by CDK12. Together, our results indicate that the phosphorylation status of all these sites depends on CDK12 catalytic activity. 

### 3.2. Sites Directly Phosphorylated by CDK12

As just mentioned, affected phosphorylation sites that resemble the CTD consensus sequence ([Y_1_S_2_P_3_T_4_S_5_P_6_S_7_]_n_) would seem the most likely to be directly phosphorylated by CDK12, which prefers to add a phosphoryl group to the S_2_ or S_5_ position (e.g., [3] [6,35]). Note that both S_2_ and S_5_ are part of an SP dipeptide (the S can also be a T residue); thus, CDK12 and its relatives are said to be ‘proline-directed’ kinases. Among the peptide sequences in Supplementary File I, Table S5, we found 11 in which the affected Ser or Thr was part of an (S/T)P dipeptide [ ‘(S/T) P’ col.] (two more were found, but they were also found in the control experiment (see below, and Supplementary File II) and were thus excluded). The 11 affected sequences are listed in Figure 2A in decreasing order of phosphate loss after treatment with inhibitory analogue 1-NM-PP1 (‘Fold ↓’ column); sequences are aligned on the affected residue, in red, with neighboring P in bold. 

With this set of sequences we used <weblogo.berkeley.edu> to find a sequence logo potentially representing a CDK12 consensus phosphorylation site. The resulting logo (Figure 2B) is of course dominated by the pre-selected ‘(S/T) P’ motif, but it shows some preference for a Proline two residues N-terminal from the (S/T) and a very weak preference for a Proline six residue N-terminal. Another preference is a hydrophobic residue (LVI) 3 positions C-terminal to the (S/T); and a weaker one is Pro or Met six positions C-terminal to the (S/T). Thus, by this analysis a conclusion might be that a CDK12 consensus phosphorylation site is Px(S/T)PxZxx(P/M) (where Z is hydrophobic). This sequence aligns well with the canonical CTD consensus if the S/T is placed at position 5 in the CTD heptad (Figure 2B, *bottom*). 

Perhaps the most likely CDK12 substrate is TPR, a component of the “basket” of the nuclear pore complex (NPC) [36], that contains three affected (S/T)P sites, with the most-affected one matching the sequence logo in Figure 2 [Px(T)PxLxxP]. Phosphorylation at this site (T2137) decreased > 5-fold during the 30 min of CDK12 inhibition, while phosphorylation at the other sites decreased around 3-fold. TPR, the ortholog of yeast Mlp1, is intriguing as a possible CDK12 substrate, since one of its roles is to prevent nuclear export of incompletely-spliced mRNAs [37,38,39]. If TPR is actually a CDK12 substrate, our results establish a connection between CDK12 and post-splicing quality control (“QC”) in addition to the previous connections made between CDK12 and splicing events *per se*. 

Another likely substrate is LIMD1, which contains two CTD-similar phosphorylation sites showing reductions after CDK12 inhibition of ~5× and 3×, respectively. The most-affected site matches the sequence logo quite well [Px(S)PxVxxP]. The LIMD1 protein functions in a number of processes, including transcriptional repression of several signaling pathways and formation of P-bodies. The repertoire of direct CDK12-catalyzed phosphorylations will interestingly increase if LIMD1 is a true substrate. 

The peptide from ADAM17 decreases 5.3-fold, and it shows a good match to the sequence logo, except that it has a less-favored Arginine 3 positions C-terminal to the phosphorylated T residue [PxTPxRxxP]. The other peptides in Figure 2A show variably less similarity to the sequence logo, but some or all of them may still be CDK12 substrates. 

### 3.3. Control for Off-Target Effects of Inhibitory Analog 1-NM-PP1

The above results suggest that the phosphorylation states of the P-peptides that decrease in abundance after 1-NM-PP1 addition depend on the catalytic activity of CDK12. To check this idea, we sought to determine whether some of the results might represent inhibition of other kinases by 1-NM-PP1 (e.g., “off-target” effects of the analog). Toward this end, we repeated the experiment of Figure 1, except using HeLa cells expressing only wildtype (WT) CDK12, an enzyme not inhibited by 1-NM-PP1 [3]. Thus, triplicate cultures of CDK12^WT^ cells were treated for 30 min with DMSO alone or with 1-NM-PP1 in DMSO, then cells were collected and extracts prepared as before. A phosphor-proteomics analysis of the CDK12^WT^ samples identified 6,295 total P-peptides corresponding to 2201 phosphoproteins (Supplementary File II, Table S4), numbers that are similar to those from the earlier CDK12^as^ analysis. P-peptides differentially represented in the CDK12^WT^ samples (minus and plus 1-NM-PP1) were defined as those whose abundance changed >2-fold (with a *p*-value <0.05) in the presence of the analog. There were 68 P-peptides that decreased >2-fold after 1-NM-PP1 addition and 3 that increased >2-fold (Supplementary File II, Table S5), again numbers similar to the CDK12^as^ analysis. 

To see if any of the decreasing P-peptides identified in the CDK12^as^ experiment also decreased in the CDK12^WT^ experiment, we looked for P-peptides that changed >2-fold in both experiments; we found 6 in-common P-peptides that decreased and 1 in-common P-peptide that increased (Table 2. These peptides are labeled as “Off Target” in Supplementary File I, Table S5). The 6 decreasing P-peptides observed in both experiments were from the following human proteins: ANLN, NF1, AHNK, HDAC4, CB044, and RAB34; a single increasing P-peptide in common was from AFAP1 (Table 2). Because the six P-peptides decreased after 1-NM-PP1 treatment even when there was no analog-sensitive CDK12 to be inhibited, we propose that their phosphorylation status depends on (wildtype) kinases in the CDK12^WT^ cells that are unintentionally inhibited by 1-NM-PP1. Notably, the decreases for the six peptides were mostly modest (Table 2. WT experiment = green highlights; analog-sensitive experiment = gray highlights), with all P-peptide reductions, except one, being less than 3-fold in the WT experiment; this may suggest only partial inhibition of off-target kinases. Moreover, none of the off-target peptides is a close match to consensus CTD repeat sequence. To identify protein kinases potentially responsible for phosphorylating the peptides in Table 2, we queried phospho-protein databases (<phosphonet.ca>; <phosphosite.org>) using the amino acid sequences of the in-common peptides. This approach implicated several enzymes including PIM1/3 and JNK1/3, along with some others, as off-target kinases potentially inhibited by 1-NM-PP1 (Table 2). 

To obtain a broader view of potential off-target analog–sensitive kinases, we queried the phospho-protein databases using all the peptides that decreased >3× in the CDK12^WT^ experiment (Supplementary. File II, Table S5). The potential kinases are listed in Table 3, ranked by the number of peptides they were implicated in phosphorylating. Prominent kinases potentially inhibited by 1-NM-PP1 again included JNK and PIM kinases, along with PRKG1/2, CDK2/3, MAPKAPK3/2, mTOR and PLK3. 

Previous work identified a number of WT kinases inhibited in vitro by 1-NM-PP1 [40,41], and we compared our in vivo-implicated kinases to those. Interestingly, our candidates mostly were either not in the previous lists or were not strongly inhibited in vitro. One explanation is that we are observing both direct as well as indirect effects of 1-NM-PP1 inhibition. For example, if a MAP kinase such as ERK2 is inhibited by the analog, a secondary effect could be a reduction in activity of a MAP kinase-activated protein kinase (e.g., MAPKAPK2). In any case, our results complement the earlier tests and highlight the possibility that off-target events may occur in experiments using inhibitory analogs such as 1-NM-PP1, and experimenters should be aware of this possibility. 

## 4. Discussion

We have identified 40 HeLa cell proteins that undergo a 2- to >12-fold loss of phosphate groups from specific sites within 30 min of adding a selective inhibitor of CDK12^as^ to the growth medium. The normal phosphorylation states of all of these proteins are thus dependent on the presence of active CDK12 kinase, and several of the proteins are likely to be direct substrates of CDK12/CyclinK. Modulation of CDK12 activity may therefore regulate processes or pathways in which these proteins are involved, notably mRNA nuclear export, signaling & transcription regulation, pre-mRNA processing, mitosis & cell division, and certain DNA transactions. 

Our results reveal several prospective protein substrates of CDK12/CyclinK beyond the CTD of RNAPII (RPB1). Perhaps the most likely direct substrate is the protein TPR (ortholog of budding yeast Mlp1), which contains three CTD-similar sites at which phosphorylation decreases rapidly after inhibition of CDK12 activity (Figure 2). TPR (yMlp1) is a 270 kDa protein that is a main component of the intranuclear filaments attached to the inner surface of nuclear pore complexes (NPCs) and is required for mRNA export. Additional connections between nuclear mRNA export and CDK12 include the observation that factors involved in nuclear export were found to co-purify with CDK12 as assessed by immunopurification approaches [3]. Similarly, five components of the Exon Junction Complex (EJC), which plays roles in quality control of splicing and in nuclear export, also co-purified with native CDK12 (RBM8A/Y14, MAGOH, EIF4A3, PININ, & ACIN1 [3]). Moreover, additional proteins in Table 1 (and Supplementary File I, Table S5) function in mRNA export from the nucleus, notably NUP214 (yNup159) as well as ZC11A. Remarkably, TPR (yMlp1) plays a role in preventing export of unspliced mRNAs [37,39], suggesting functional links between different subsets of CDK12-connected proteins. Interestingly, the yeast ortholog of CDK12 (Ctk1) also has been implicated in an export pathway that includes the ortholog of TPR (yMlp1) [42]. Thus, diverse experimental approaches implicate CDK12 not only in pre-mRNA processing itself but also in quality control of that processing, with our phospho-proteomics results connecting CDK12 to TPR & Nup214 and thus to mRNA nuclear export. We propose that multiple protein-protein interactions, mediated in part by CDK12 and the phosphate groups it deposits, generate a series of complexes (or a mega-complex) in which RNA polymerase, components of the RNA processing pathways, and the mRNA export/NPC machinery interact to shuttle transcripts through the “mRNA maturation” pathway. 

Unexpectedly, the phospho-peptide that decreased the most after CDK12 inhibition (>12-fold) was from the DNA damage repair protein XPC. While the two affected phosphorylation sites in this XPC peptide are extremely different from the consensus CTD repeats (see Supplementary File I, Table S5), it will still be interesting to determine whether CDK12 directly phosphorylates either of these sites. In any event, connecting XPC phosphorylation to CDK12 is intriguing for a number of reasons. For one, Tjian and colleagues have shown that XPC (as part of the ‘stem cell coactivator’ (SCC) complex that functions as a transcriptional activator [43]), is necessary to maintain the non-differentiated state of mouse embryonic stem cells (mESC). For another, Li and colleagues previously showed that CDK12 itself is required to maintain the undifferentiated state of mESC cells [22]. One might speculate that CDK12-dependent phosphorylation of XPC is the link tying these two observations together.

Among previously identified CDK12-associating proteins were some that interact with the 5′ cap-end of RNAPII-synthesized RNAs, and in an earlier paragraph we focused on the nuclear roles of these proteins, including mRNA nuclear export. On the other hand, these multi-functional proteins are perhaps better known for their roles in translation. Intriguingly, recent results from Jones and colleagues also connect CDK12 with proteins that bind the cap-end of RNAPII transcripts (notably the cap-binding repressor 4E-BP1), and in this case the result of CDK12 activity is in fact regulation of translation [31]. After CDK12 depletion, translation of a subset of mRNAs is decreased, and a number of these mRNAs encode subunits of mitotic and centromere/ centrosome complexes. Consistent with this finding, CDK12 depletion leads to major defects in chromosome alignment and in progression through mitosis. These effects are congruent with our previous finding that inhibiting CDK12 activity impedes cell proliferation [5] and with our current finding that several mitosis-related proteins are phosphorylated in a CDK12 activity-dependent manner (e.g., Table 1). 

Another likely substrate of CDK12 is LIMD1, with two CTD-similar phosphorylation sites significantly affected by CDK12 inhibition (Figure 2). LIMD1 is a multifunctional protein involved in, among other things, the assembly of numerous protein complexes, repression of gene transcription, cell proliferation, and cell migration. One way it participates in the regulation of transcription is by acting as a transcriptional co-repressor. Moreover, LIMD1 is also essential for P-body formation and integrity. While it is not clear which of its functions may depend on phosphorylation at the two potential CDK12 sites shown in Figure 2, it is worth pointing out that phosphorylation at Ser424 decreases over 5-fold after just 30 min in the presence of the CDK12^as^-inhibitory analog, suggesting an important in vivo role for this phosphorylation event.

Other proteins in the signaling/Txn-regulation group also show significant phosphate loss after CDK12 inhibition, but several of the sites involved do not resemble the CTD at all, such as those in MK03 and MK01 (known to be phosphorylated by MEK1/2), RGH35, ANS1A, KANK2 and BCAR3 (cf. Supplementary. Data I, Table S5). We propose that, in some way yet to be determined, inhibiting CDK12 activity results in inactivation of kinases, or activation of phosphatases, influencing these phosphorylation sites.

Two other proteins, both with P-peptides showing a modest 2.2-fold decrease in amount after CDK12 inhibition (Supplementary File I Table S5), are worth pointing out because they have other connections to CDK12 or transcription elongation. One is MYPT1 (PPP1R12A), a regulatory subunit of the phosphatase PPP1, that was found to co-purify with native CDK12 [3]. The association of PPP1R12A with CDK12 might suggest a substrate/kinase relationship, but the affected P-site (underlined) in PPP1R12A (LASTSDIEEK) does not obviously resemble the CTD repeats. Another is NELFE, a subunit of transcription elongation factor NELF; again, however, the P-site (underlined) in NELFE (SISADDDLQESSR) is not CTD-like.

Finally, to explore the possible influence of “off-target” effects of 1-NM-PP1 on the phospho-proteomics results, we carried out a control experiment utilizing cells expressing only wildtype CDK12^WT^ (which is not inhibited by 1-NM-PP1 - see [3]). This experiment revealed that the analog actually does modulate the phosphorylation state of a number of peptides from different proteins. Because these phosphorylation changes occur even in the absence of an analog-sensitive CDK12^as^, they are presumably caused by 1-NM-PP1 acting on off-target protein kinases. Relevantly, previous work had identified several wildtype protein kinases inhibited by 1-NM-PP1 in vitro [40,41]. Interestingly, the kinases implicated by our in vivo results are mostly different from those identified earlier. We speculate that the P-peptide changes we observed represent both primary and secondary (downstream) effects of inhibiting off-target kinases. In any event, the P-peptides we identified as changing significantly after adding 1-NM-PP1 to “WT” cells (Table 2 and Table 3, and Supplementary File II Table S5) represent a potential “background” that might appear in other experiments in which 1-NM-PP1 is utilized to inhibit a mutationally-sensitized enzyme in living cells.

## 5. Conclusions

We previously showed that inhibiting CDK12 activity in living cells leads rapidly to altered phosphorylation states of the RNAPII CTD, and we now demonstrate that CDK12 inhibition also alters phosphorylation states of several dozen other proteins. From inspecting the amino acid sequences of the affected phosphorylation sites, we conclude that some are phosphorylated directly by CDK12, while others are phosphorylated by different kinases and are affected indirectly by CDK12 inhibition; however, the normal phosphorylation levels at all of the sites depend on CDK12 catalytic activity. Instructively, a number of CDK12-dependent phosphorylation events affect proteins involved in pre-mRNA processing and mRNA nuclear export, including nuclear pore complex components; these observations lead us to propose that CDK12 plays a role in functionally bridging between RNA processing machineries and the nuclear pore complex. In addition, CKD12 inhibition leads to dramatic dephosphorylation at a site in the XPC protein. Since both CDK12 and XPC are required for maintenance of the undifferentiated state of mouse embryonic stem cells, we speculate that this double requirement involves the CDK12-dependent XPC phosphorylation. Among the other phosphorylation events that depend on CDK12 activity are several that affect proteins involved in mitosis, and we surmise that defective phosphorylation of these proteins in CDK12-deficient ovarian and prostate cells contributes to tumor development. We hope that future use of the CDK12as cell line will provide a better understanding of these and other CDK12-dependent events, elucidating specific in vivo functions of CDK12 and providing new insights into its role as a tumor suppressor.

## Figures and Tables

**Table 1:** Proteins yielding phospho-peptides that decrease > 3-fold after CDK12 inhibition. Proteins that produced phospho-peptides decreasing 3-fold or more after 30 min of 1-NM-PP1 treatment are grouped in five broad functional categories described by the terms in the “Keywords” column. Within a category, the proteins are listed in decreasing order of the fold change of the P-peptide(s) produced; peptide sequences are presented in Supplementary File I, Table S5. TPR and AHNK produced >1 P-peptide each (Comments column). Colors indicate functional categories (yellow = transcription regulation/signaling; green = mRNA maturation; orange = cell division/mitosis; blue = DNA transactions).

Protein Name	Protein Description	Gene Name	Fold Change	Functional “Keywords”	Comments
MK03_HUMAN	Mitogen-activated protein kinase 3	MAPK3	−6.3	signaling; Txn-regulation	
SOS1_HUMAN	Son of sevenless homolog 1	SOS1	−6.1	signaling	
RHG35_HUMAN	Rho GTPase-activating protein 35	ARHGAP35	−5.6	Txn-regulation; signaling	
LIMD1_HUMAN	LIM domain-containing protein 1	LIMD1	−5.4	Txn-regulation; scaffold prot; cell fate	
ANS1A_HUMAN	Ankyrin repeat and SAM domain-containing protein 1A	ANKS1A	−4.2	signaling; cell migration	
KANK2_HUMAN	KN motif and ankyrin repeat domain-containing protein 2	KANK2	−4.0	Txn-regulation; steroid co-activators	
MK01_HUMAN	Mitogen-activated protein kinase 1	MAPK1	−3.7	signaling; Txn-regulation	
BCAR3_HUMAN	Breast cancer anti-estrogen resistance protein 3	BCAR3	−3.1	signaling; reg. DNA synthesis	
NU214_HUMAN	Nuclear pore complex protein Nup214	NUP214	−5.3	mRNA maturation: nuclear export	
TPR_HUMAN	Nucleoprotein TPR	TPR	−5.1	mRNA maturation: nuclear export	TPR, pep 1
AHNK_HUMAN	Neuroblast differentiation-associated protein AHNAK	AHNAK	−4.9	mRNA maturation: poly A binding; splicing	AHNK, pep 1
DDX20 / Gemin 3	Probable ATP-dependent RNA helicase DDX20	DDX20	−4.2	mRNA maturation: splicing; snRNP	
AHNK_HUMAN	Neuroblast differentiation-associated protein AHNAK	AHNAK	−3.6	mRNA maturation: poly A binding; splicing	AHNK, pep 2
AHNK_HUMAN	Neuroblast differentiation-associated protein AHNAK	AHNAK	−3.5	mRNA maturation: poly A binding; splicing	AHNK, pep 3
TPR_HUMAN	Nucleoprotein TPR	TPR	−3.2	mRNA maturation: nuclear export	TPR, pep 2
PARD3_HUMAN	Partitioning defective 3 homolog	PARD3	−11.1	cell division; signaling	
SEPT7_HUMAN	Septin-7	SEPT7	−6.3	mitosis; kinetochore; ciliogenesis	
ADA17_HUMAN	ADAM 17	ADAM17	−5.3	mitosis; signaling; spindle	
CLAP2_HUMAN	CLIP-associating protein 2	CLASP2	−3.4	microtubule-binding; kinetochore	
XPC_HUMAN	DNA repair protein complementing XP-C	XPC	>−12	DNA repair; Txn	
DPOLA_HUMAN	DNA polymerase alpha catalytic subunit	POLA1	−3.0	DNA synthesis	
CTNA1_HUMAN	Catenin alpha-1	CTNNA1	−6.1	other… cell adhesion	
ARFP1_HUMAN	Arfaptin-1	ARFIP1	−5.2	other… Golgi; vessicles; phospholipase D	

**Table 2:** Peptides decreasing >2-fold in both experimental (CDK12^as^ = ‘as’) and control (CDK12^WT^ = ‘WT’) experiments. Phosphorylated residue indicated in the ‘Peptide’ column.

Protein	Peptide	Fold Change		Potential Protein Kinase
		*‘as’*	*‘WT’*	
**HDAC4**	SSPLLR (S266)	−2.7	−5.8	**PIM1; PRKG1; PIM3**
**AHNK**	VSMPDVELNLKSPK (S3426)	−3	−2.7	**JNK1/3; P38d (MAPK13)**
**ANLN**	TQSLPVTEK (S485)	−4.3	−2.5	**ANPa; COT; ANPb;**
**RAB34**	INSDDSNLYLTASK (S241)	−2.5	−2.4	**MAPKAPK3; ANPa; PRKG2**
**NF1**	SFDHLISDTK (S2543)	−4.1	−2.2	**PIM1/3; MSK1**
**WDCP**	DSFSHSPGAVSSLK (S690)	−2.6	−2	**ERK1/2/5**
**AFAP1**	LSSERPSSDGEGVVE… (S283)	(+)2.1	(+) 3.4	**CK2a2 (CSNK2A2); CK2a1 (CSNK2A1); MAPKAPK3**

**Table 3:** Kinases potentially inhibited by analog 1-NM-PP1.

Potential Kinase	# Sites
PIM 1, 2 or 3	5
PRKG1 or 2	5
CDK2 or 3	3
JNK1 or 3	3
MAPKAPK3 or 2	3
mTOR/FRAP	2
PLK3	2
BARK2/1	1
CAMK4	1
CDK7	1
CDK9	1
CHK2	1
GSK2B	1
GSK3A	1
IKKb	1
PFTAIRE2	1
RHOK	1
ROCK1	1
SCYL2	1

## Supplementary Materials

The following are available online at https://www.mdpi.com/2218-273X/9/10/634/s1, File SI: Phosphoproteomic Profiling: CDK12 Analog-Sensitive Cells, File SII: Phosphoproteomic Profiling: CDK12 WT Cells.
